# Strategic enforcement of linear payoff relations in a three-player strictly alternating prisoner’s dilemma game

**DOI:** 10.1038/s41598-025-32002-0

**Published:** 2026-02-01

**Authors:** Mohammad A. Taha, Samira S. Mersal, M. G. Brikaa

**Affiliations:** 1 Department of Mathematics, Faculty of Science, Suez Canal University, Ismailia, 41522 Egypt; 2 Department of Basic Sciences, Faculty of Computers and Informatics, Suez Canal University, Ismailia, 41522 Egypt

**Keywords:** Game theory, Strictly alternating prisoner’s dilemma, Zero-determinant strategies, Three-player game, Evolution, Mathematics and computing, Physics

## Abstract

The prisoner’s dilemma game is a fundamental model in game theory for studying the emergence of cooperation among self-interested individuals with conflicting incentives. Most existing studies have examined the simultaneous two-player version, where both players act simultaneously. In contrast, this paper investigates the impact of Zero-Determinant strategies in a strictly alternating three-player repeated model of the prisoner’s dilemma game, where players take turns making decisions based on the previous actions of their opponents (one-memory strategy). Analytical results reveal that the Zero-Determinant strategies in the strictly alternating model differ significantly from those in the simultaneous three-player prisoner’s dilemma game. We further examine the equalizer and extortion subsets of Zero-Determinant strategies and derive their feasible regions within this framework. These findings provide new insights into the strategic control and cooperation mechanisms in multi-player alternating interactions.

## Introduction

In game theory, the Prisoner’s Dilemma (PD) is one of the most well-known models for analyzing cooperative behavior among rational individuals. It has attracted significant attention because it illustrates how two perfectly rational players may choose not to cooperate, even when mutual cooperation would yield a better outcome for both.

Most previous studies have been concerned with the simultaneous model of the PD game, in which each player selects a strategy without knowing the other’s choice. Nowak et al.^1^ investigated the simultaneous Iterated Prisoner’s Dilemma (IPD) game using two-state automata, where the classic game is repeated between the same players. They determined the payoff matrix for the strategies of this game. In this model, both players are assumed to make their decisions simultaneously in each round. IPD game consists of multiple repetitions of the one-shot PD, allowing players to adjust their actions based on previous interactions. This means that a strategy for the IPD is a mapping from any previous rounds of this game into what to do in the next round. The entire picture changes substantially when the IPD game is played over an extended period of time with finite memory of past states, as previously examined in studies^2–5^.

Nowak and Sigmund^6^ examined several models of the IPD game, including alternating models (randomly and strictly alternating PD games). In the alternating model, the participants take turns making their actions, as previously discussed in studies^7–9^. This assumption can lead to a strictly alternating game, in which the players always take their actions in turns, or to a stochastically alternating model, where in each round the player to take action is selected at random and the next is selected probabilistically.

The alternating model is very important for characterizing many biological situations. Using vampire bats as an example, a bat that has discovered food would share it with another that has not, which is clearly not modelled as a simultaneous exchange^10^. “Predator Inspection” is another example that is often used. In this case, two fish approach and inspect a potential predator, even though it is safer for both fish to stay back and observe the other’s fate. Even though their behavior appears to be coordinated, there is no evidence that they make simultaneous judgments as opposed to quick but asynchronous ones^11,12^.

Building upon these sequential interaction models, recent advances have focused on strategic control mechanisms within repeated games, particularly the emergence of Zero-Determinant (ZD) strategies. Although game theory and the PD game have been extensively studied for decades, the introduction of Zero-Determinant strategies by Press and Dyson^13^ marked a remarkable breakthrough. Their discovery reignited interest in the PD, particularly in evolutionary biology and computer science^14–19^. Taha and Ghoneim^20^ extended this framework to infinitely repeated asymmetric two-player games, identifying the feasible regions of the studied strategies in these games. Ueda^21^ demonstrated that the Tit-For-Tat (TFT) strategy in an infinitely repeated two-player game becomes unbeatable if it is a ZD strategy. McAvoy and Hauert^22^ further generalized these ideas by introducing autocratic strategies, showing that ZD strategies exist in both strictly and randomly alternating PD games. Park et al.^23^ later examined alternating games with memory-one strategies, extending the model through evolutionary simulations and exploring the effects of non-regular alternation patterns and longer memory depth.

Although existing ZD analyses have largely been confined to two-player frameworks, many real-world cooperative dilemmas involve interactions among three or more agents. In such multi-agent environments, pairwise reciprocity alone cannot explain the information of coalitions, indirect retaliation, or the diffusion of cooperative norms. Extending the PD game to a strictly alternating three-player setting therefore allows us to capture richer behavioral dynamics and identify whether ZD control principles persist when strategic interdependence increases. This extension also provides new theoretical insight into how cooperation scales beyond dyadic relationships, revealing novel equilibrium structures that do not appear in two-player models.

In this paper, we extend the analytical framework introduced of Press and Dyson in^13^ to a strictly alternating three-player PD, where players act sequentially rather than simultaneously. This generalization not only expands the class of games where ZD strategies can be characterized but also deepens our understanding of how alternating decisions affect strategic enforcement and fairness in multi-agent systems. We are seeking to examine the emergence of ZD (equalizer and extortion) strategies in the proposed game. For the strictly alternating three-player PD game, we analytically derived the feasible regions of the considered ZD strategies, focusing on single-unit memory strategies and an infinitely repeated game. Single-unit memory means that each player takes his action in a round, and the three consecutive rounds represent a unit. Our analysis follows the approach of Taha and Ghoneim^24^, with the key difference that in this study, the focal player (ZD player) has two distinct mechanisms for enforcing ZD strategies.

The paper will be ordered as follows. The next section will discuss two models for a two-player IPD game. “The strictly alternating $2\times2$ PD game” section illustrates the model of the $2\times2$ strictly alternating PD game, while “ZD strategies in SAPD $2\times2$ game” section illustrates the ZD strategies in the $2\times2$ strictly alternating PD game. “Game formulation” section illustrates the three-player strictly alternating PD game. The feasible regions of the ZD strategies subsets (equalizer and extortion) for the three-player strictly alternating PD model are mathematically deduced in “Repeated three-player SAPD game: ZD strategies” section. In “Numerical example” section, we have presented a numerical example to clarify the main idea of the proposed model. Finally, in “Conclusion” section, the findings are summarized, and the future work is discussed.

## The different models of the PD game

The classic PD game is a two-person game and there are two choices available to each player, cooperate ($\:C$) or defect ($\:D$). Although the two players know that cooperation is the most effective outcome, this game explains the reasons why two rational players may not be able to work together. If the two players choose to cooperate, they will get a reward $\:R$; if the two players defect, they will get a punishment payoff $\:P$; and when one player defects and the other choose to cooperate, the defecting player will get a temptation payoff $\:T$, while the cooperating player will receive a sucker payoff $\:S$. These values are constrained by the inequalities $\:T>R>P>S$ and $\:2R>T+S$. According to the last inequality, always cooperating is more advantageous than alternately cooperating and defecting. Due to the symmetric nature of the game, $\:T,\:R,\:P$, and $\:S$ are identical for the two players. The payoff matrix of PD game is shown in Table 1.

**Table 1:**
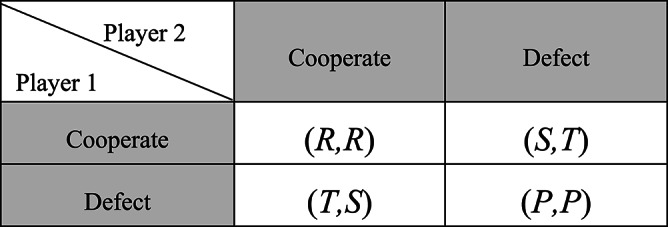
The payoff matrix of PD game.

This game has many models according to the order of taking actions. If the two players take their actions at the same time, then the model will be the simultaneous one. If the players take their actions sequentially, then it will be the alternating model.

### The model of alternating $2\times2$ PD game

In the model of alternating prisoner’s dilemma (APD) game, we have two players with two options for each one. The player can either to cooperate $\:\left(C\right)$ or defect $\:\left(D\right)$ with the other player. In contrast to the simultaneous model, the symmetry in the alternating one between the two players will be broken. The reason for this difference from the simultaneous model is that in each round, one of the players is considered to be the leader and can decide the payoff of this round, furthermore each player has the ability to know the other player’s choice before he takes his own action. If the leader chooses to cooperate $\:C$ with his opponent, then he receives payoff $\:\boldsymbol{a}$ and the second player receives payoff $\:\boldsymbol{b}$. The other choice for the leader $\:D$ will give him payoff $\:\boldsymbol{c}$ and the other player $\:\boldsymbol{d}$. Let us assume the following conditions for this game

1 $${\boldsymbol{c}}>{\boldsymbol{a}}\;{\mathrm{~and~}}\;0<{\boldsymbol{c}} - {\boldsymbol{a}}<{\boldsymbol{b}} - {\boldsymbol{d}}$$

The first condition of Eq. (1) means that the action $\:D$ is more beneficial for the leader player. The other condition states that the cost to the leader player according to the cooperation behavior is less than the benefit to the other one. Let us consider that two successive rounds represent one unit of payoff, then if two players choose $\:C$ they obtain $\:\boldsymbol{a}+\boldsymbol{b}$ while if both players choose $\:D$ they obtain $\:\boldsymbol{c}+\boldsymbol{d}$. When one player selects $\:C$ and the other opts for defection $\:D$, then the cooperator receives $\:\boldsymbol{a}+\boldsymbol{d}$, while the defector obtains $\:\boldsymbol{c}+\boldsymbol{b}$ as shown Table 1. These payoffs for two successive rounds are similar to the payoffs of the simultaneous model of PD game, and it can be denoted by $\:T=\boldsymbol{c}+\boldsymbol{b},\:R=\boldsymbol{a}+\boldsymbol{b},\:P=\boldsymbol{c}+\boldsymbol{d}$ and $\:S=\boldsymbol{a}+\boldsymbol{d}$.

Referring to inequality in Eq. (1) and the values of payoff for the two successive rounds in APD, we have $\:T>R>P>S$, $\:2R>T+S$ and $\:T+S=R+P$. The first two conditions are similar to the conditions of simultaneous mode of this game. In this study, we are concerned only with the strictly alternating model of PD game.

## The strictly alternating $2\times2$ PD game

**Fig. 1:**
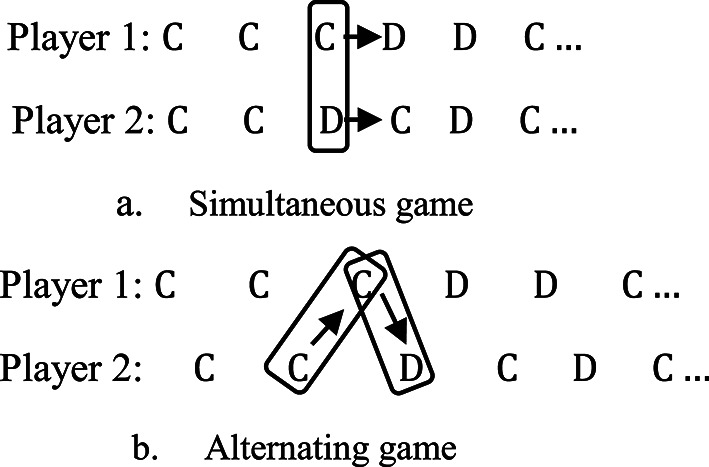
The transitions among states in simultaneous and alternating games.

Here, we shall consider the infinitely iterated strictly alternating PD (SAPD) game. In the (SAPD) model, each player takes an action in turn. One complete interaction between the two players (two consecutive rounds) is regarded as a single unit. A memory-1 strategy in this setting means that each player’s current decision depends on the outcomes of the previous interaction unit. Suppose that one player has the strategy $\:\boldsymbol{p}=({p}_{1},{p}_{2},{p}_{3},{p}_{4})$ and the other player opts the strategy $\:\boldsymbol{q}=({q}_{1},{q}_{2},{q}_{3},{q}_{4})$ and the payoffs $\:T,R,P$ or $\:S$ will be numbered from 1 to 4. To determine the transition matrix among the states of the game, let us denote $\:{p}_{i}$ to be the possibility of choosing $\:C$ after the outcome $\:i$ in the previous round and similarly $\:{q}_{i}$ of the other player. In the alternating games, the players respond to the last two recent rounds (representing one unit) because they play with memory-one strategies. Figure 1 shows the difference between the transitions among the states in the two cases. The rectangle around the actions represents one unit.

We can obtain the transition matrix of this game from round to another round by Markov chain as the following

2 $$\:M(\boldsymbol{p},\boldsymbol{q})\:\:\:=\left[\begin{array}{llll}{p}_{1}{q}_{1}&\:{p}_{1}(1-{q}_{1})&\:(1-{p}_{1}){q}_{2}&\:(1-{p}_{1})(1-{q}_{2})\\\:{p}_{2}{q}_{3}&\:{p}_{2}(1-{q}_{3})&\:(1-{p}_{2}){q}_{4}&\:\left(1-{p}_{2}\right)\left(1-{q}_{4}\right)\\\:{p}_{3}{q}_{1}&\:{p}_{3}(1-{q}_{1})&\:(1-{p}_{3}){q}_{2}&\:(1-{p}_{3})(1-{q}_{2})\\\:{p}_{4}{q}_{3}&\:{p}_{4}(1-{q}_{3})&\:(1-{p}_{4}){q}_{4}&\:(1-{p}_{4})(1-{q}_{4})\end{array}\right]$$

The rows of the matrix in Eq. (2) represent the sates $\:CC,\:CD,\:DC,$ and $\:DD$, respectively and similarly the columns of this matrix. For example, moving from the state $\:DC$ to the state $\:CD$ will be $\:{p}_{3}(1-{q}_{1})$, where $\:{p}_{3}$ is equal to the probability for player 1 to cooperate after outcome $\:DC,$ and $\:(1-{q}_{1})$ is equal to the probability for player 2 to defect after outcome $\:CC$.

In this symmetric setting, the ordering of states differs depending on which player initiates the move. Specifically: for player 1, the sequence of states is $\:\{CC,CD,DC,DD\}$ has the stationary distribution $\:\pi\:=({\pi\:}_{1},{\pi\:}_{2},{\pi\:}_{3},{\pi\:}_{4})$. For player 2, the corresponding ordering becomes $\:\{CC,DC,CD,DD\}$ has the stationary distribution $\:\pi\:=({\pi\:}_{1},{\pi\:}_{3},{\pi\:}_{2},{\pi\:}_{4})$, since the roles of the players are interchanged.

The matrix $\:M$ in Eq. (2) represents the transition matrix of player 1 when he moves first. Using the same procedure, we can likewise compute the transition matrix for the case in which player 2 moves first.

If the components of the strategies $\:\boldsymbol{p}$,$\:\boldsymbol{q}>0$, then $\:M$ has a unique left eigenvector $\:\boldsymbol{\pi\:}=({\pi\:}_{1},{\pi\:}_{2},{\pi\:}_{3},{\pi\:}_{4})$ to the eigenvalue 1 with the components of $\:\boldsymbol{\pi\:}$ are greater than zero and $\:\sum\:{\pi\:}_{i}=1$. The expected payoff of player 1 opting $\:\boldsymbol{p}$ in this game can be given by $\:{E}_{1}=\:\boldsymbol{\pi\:}.\:{{\Omega\:}}_{1}={\pi\:}_{1}R+{\pi\:}_{2}S+{\pi\:}_{3}T+{\pi\:}_{4}P$ and for the player with the strategy $\:\boldsymbol{q}$ is given by $\:{E}_{2}=\:\boldsymbol{\pi\:}.\:{{\Omega\:}}_{2}={\pi\:}_{1}R+{\pi\:}_{2}T+{\pi\:}_{3}S+{\pi\:}_{4}P$ ,where $\:{{\Omega\:}}_{1}=(R,S,T,P)$ and $\:{{\Omega\:}}_{2}=(R,T,S,P)$ are the payoff vectors of player 1 and player 2, respectively. The vector $\:\boldsymbol{\pi\:}$ denotes the stationary distribution vector of outcomes when the game is played infinitely, then it can be determined by the following Eq. 

3 $$\boldsymbol{\pi }M=\boldsymbol{\pi }$$

This is because the unique left eigenvector is also the unique limiting distribution, and the players’ payoffs are given by the limit of means.

## ZD strategies in SAPD $2\times2$ game

The purpose of this section is to explore the effect of ZD strategies on the repeated SAPD game. The ZD strategies in asynchronous PD game have been discussed by Young^25^, and he supposed that a sequential game involving two players, with player 1 moving first in the first case, and the other case he supposed that player 2 would move first. According to these two considerations, the player who moves first can employ ZD strategies. In this study, it is supposed that player 1 who moves first, and the second case where player 2 moves first can be processed similarly.

Returning to the Eq. (3), we can write it in the form

4 $$\:\boldsymbol{\pi\:}\left(M-{I}_{4\times\:4}\right)=\boldsymbol{\pi\:}{M}^{\mathbf{{\prime\:}}}=0$$

where, $\:{M}^{\boldsymbol{{\prime\:}}}=M-{I}_{4\times\:4}$, $\:0=(\mathrm{0,0},\mathrm{0,0})$ and $\:{I}_{4\times\:4}$ is the identity matrix from the same dimensions of matrix $\:M$. Now, the matrix $\:{M}^{\boldsymbol{{\prime\:}}}$ can be in the form

$$\:{M}^{\mathbf{{\prime\:}}}=\left[\begin{array}{llll}{p}_{1}{q}_{1}-1&\:{p}_{1}(1-{q}_{1})&\:(1-{p}_{1}){q}_{2}&\:(1-{p}_{1})(1-{q}_{2})\\\:{p}_{2}{q}_{3}&\:{p}_{2}\left(1-{q}_{3}\right)-1&\:(1-{p}_{2}){q}_{4}&\:\left(1-{p}_{2}\right)\left(1-{q}_{4}\right)\\\:{p}_{3}{q}_{1}&\:{p}_{3}(1-{q}_{1})&\:\left(1-{p}_{3}\right){q}_{2}-1&\:(1-{p}_{3})(1-{q}_{2})\\\:{p}_{4}{q}_{3}&\:{p}_{4}(1-{q}_{3})&\:(1-{p}_{4}){q}_{4}&\:\left(1-{p}_{4}\right)\left(1-{q}_{4}\right)-1\end{array}\right]$$

When the first column is added to the second, the result is

It is noticed that the second column is controlled by player 1 (the leader) who uses strategy $\:\boldsymbol{p}$ and the other player (the follower) has no role in this matrix. In contrast to the simultaneous model, the two players in the game have the same chance of using these strategies. This means that the leader player (who moves first) in this game is the only player who is able to use ZD strategies to determine his opponent’s payoff. Suppose that $\:\stackrel{\sim}{\boldsymbol{p}}=({p}_{1}-1,{p}_{2}-1,\:{p}_{3},{p}_{4})$, which depends only on the leader player’s strategy. By using Cramer’s rule, we have $\:Adj\left({M}^{\boldsymbol{{\prime\:}}}\right){M}^{\boldsymbol{{\prime\:}}}=\mathrm{det}\left({M}^{\boldsymbol{{\prime\:}}}\right){I}_{4\times\:4}$, and since we concern only with the non-zero solution of Eq. (4), then $\:\mathrm{d}\mathrm{e}\mathrm{t}\left({M}^{\boldsymbol{{\prime\:}}}\right)$ equal zero and $\:Adj\left({M}^{\boldsymbol{{\prime\:}}}\right){M}^{\boldsymbol{{\prime\:}}}=\mathrm{det}\left({M}^{\boldsymbol{{\prime\:}}}\right){I}_{4\times\:4}=0$, where $\:{I}_{4\times\:4}$ is the identity matrix and $\:0$ is the zero matrix. Then for any 4-dimensional vector $\:\boldsymbol{f}=({f}_{1},{f}_{2},{f}_{3},{f}_{4})$, we have $\:\boldsymbol{\pi\:} \cdot f\equiv\:D(\boldsymbol{p},\boldsymbol{q},\boldsymbol{f})$, where $\:D(\boldsymbol{p},\boldsymbol{q},\boldsymbol{f})$ is the $\:4\times\:4$ determinant given by

$$\:D\left(\boldsymbol{p},\boldsymbol{q},\boldsymbol{f}\right)=\mathrm{d}\mathrm{e}\mathrm{t}\left[\begin{array}{llll}{p}_{1}{q}_{1}-1&\:{p}_{1}-1&\:(1-{p}_{1}){q}_{2}&\:{f}_{1}\\\:{p}_{2}{q}_{3}&\:{p}_{2}-1&\:(1-{p}_{2}){q}_{4}&\:{f}_{2}\\\:{p}_{3}{q}_{1}&\:{p}_{3}&\:\left(1-{p}_{3}\right){q}_{2}-1&\:{f}_{3}\\\:{p}_{4}{q}_{3}&\:{p}_{4}&\:(1-{p}_{4}){q}_{4}&\:{f}_{4}\end{array}\right]$$

Player 1’s expected payoff is given by $\:{E}_{1}=\frac{D(\boldsymbol{p},\boldsymbol{q},{{\Omega\:}}_{1})}{D(\boldsymbol{p},\boldsymbol{q},1)}$.

Consider that player 1 use strategy $\:\stackrel{\sim}{\boldsymbol{p}}=\left({p}_{1}-1,\:{p}_{2}-1,\:{p}_{3},\:{p}_{4}\right)$, then he can implement ZD strategy by opting a strategy

5 $$\:\stackrel{\sim}{\boldsymbol{p}}=\left({p}_{1}-1,\:{p}_{2}-1,\:{p}_{3},\:{p}_{4}\right)=\alpha\:{\boldsymbol{\Omega\:}}_{1}+\beta\:{\boldsymbol{\Omega\:}}_{2}+\gamma\:1$$

If player 1 wants to use equalizer ZD strategy, he will let $\:\alpha\:=0$ in Eq. (5), then we get

$$\:\left({p}_{1}-1,\:{p}_{2}-1,\:{p}_{3},\:{p}_{4}\right)=\beta\:{\boldsymbol{\varOmega\:}}_{2}+\gamma\:1$$

**Fig. 2:**
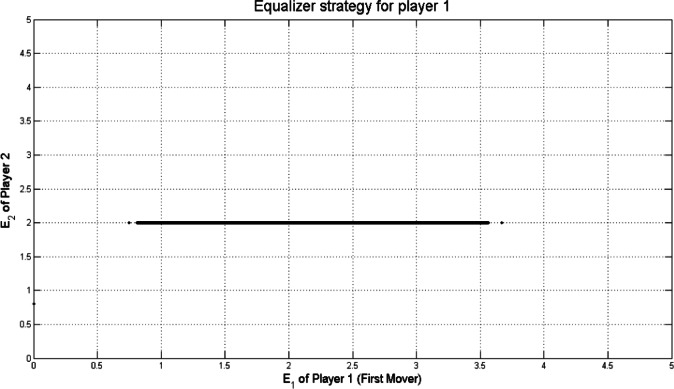
Equalizer strategy $\:\boldsymbol{p}=(\frac{2}{3},0,\frac{2}{3},\frac{1}{3})$ for player 1 (First mover) with $\:T=5,\:R=3,\:P=1,\:S=0$.

By straight forward computations as before in^13,20^, we can specify the equalizer ZD strategy for player 1. If we use $\:T=5,\:R=3,\:P=1,\:S=0$, Fig. 2 shows the equalizer strategy for player 1 against $\:14.641\times\:{10}^{3}$ uniformly sampled $\:\boldsymbol{q}$ strategies.

**Fig. 3:**
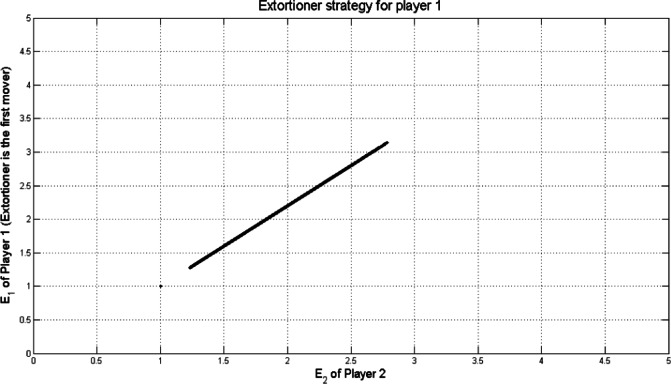
Extortion strategy $\:\boldsymbol{p}=\left(\mathrm{0.94,0.13,0.78,0}\right)$ for player 1 (First mover) with $\:T=5,\:R=3,\:P=1,\:S=0$.

If player 1 wants to use the extortion ZD strategy, he can select the strategy determined by

6 $$\:\stackrel{\sim}{\boldsymbol{p}}=\left({p}_{1}-1,\:{p}_{2}-1,\:{p}_{3},\:{p}_{4}\right)=\varphi\:\left[\left({\boldsymbol{\Omega\:}}_{1}-P1\right)-\chi\:\left({\boldsymbol{\Omega\:}}_{2}-P1\right)\right]$$

where $\:\varphi\:$ is positive parameter, and $\:\chi$ $\:\ge\:1$ is the extortion factor. By the same method discussed in^13,20^, the extortion strategy for player 1 was derived as shown in Fig. 3 assuming the following values *T* =5, *R*= 3, *P* = 1, *S* = 0 against $\:14.641\times\:{10}^{3}$ uniformly sampled $\:\boldsymbol{q}$ strategies.

**Fig. 4:**
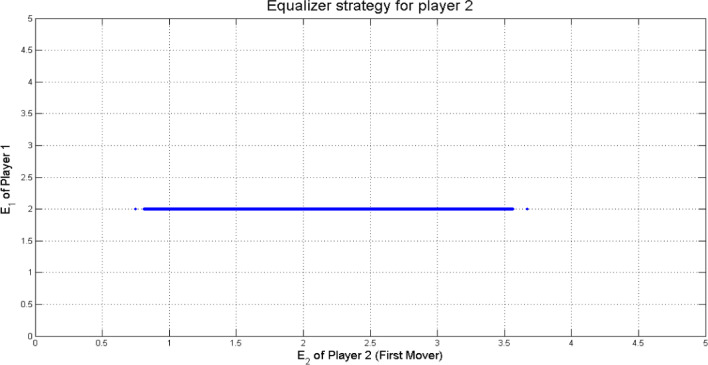
Equalizer strategy $\:\boldsymbol{q}=(\frac{2}{3},0,\frac{2}{3},\frac{1}{3})$ for player 2 (First mover) with $\:T=5,\:R=3,\:P=1,\:S=0$.

**Fig. 5:**
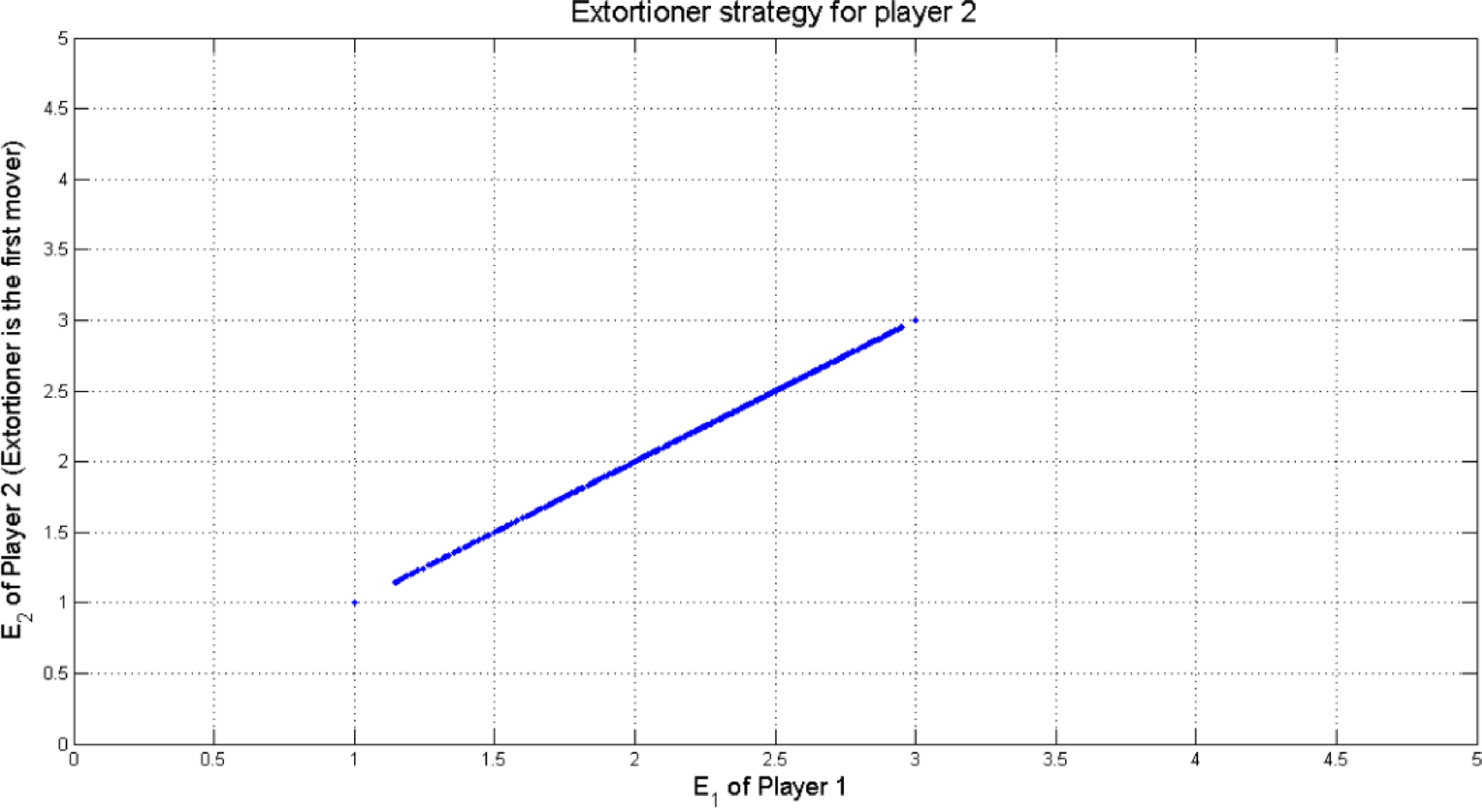
Extortionate strategy $\:\boldsymbol{q}=(\mathrm{1,0},\mathrm{1,0})$ for player 2 (First mover) with $\:T=5,\:R=3,\:P=1,\:S=0$.

If player 2 is the first mover in this game, then he can employ the equalizer and extortion strategy as player 1. Figures 4 and 5 shows the equalizer and extortionate strategy for player 2 against $\:14.641\times\:{10}^{3}$ uniformly sampled $\:\boldsymbol{p}$ strategies, respectively.

According to the symmetry of the game, each player can use ZD strategies to equalize and extortionate his opponent. It is noticed that each player has its extortion factor ($\:{\chi\:}_{1}$ for player 1 and $\:{\chi\:}_{2}$ for player 2). Due to the change in extortion factors, the value of $\:\varphi\:$ also change.

Points that appear in the Figs. 2, 3, 4 and 5 outside the main set correspond to boundary or near-boundary cases caused by rounding and discrete sampling during the simulation process. While these points do not affect the overall analytical trend, they reflect the finite precision of the computational results.

## Game formulation

The purpose of this study is to examine the ZD strategies in a 3-player SAPD game. The three players possess two identical options, “cooperation” $\:\left(C\right)$ and “defection” $\:\left(D\right)$, as well as the accompanying payoff values for each decision. Hence, the players’ identities are irrelevant. That is, a player is only concerned with whether his opponents decide to collaborate, one of them chooses cooperation and the other defection, or both choose defection, regardless of who did what. The payoff received by player 1 is influenced by the decisions made by the other players. Table 2 describes the matrix of payoff of this game.

**Table 2:**
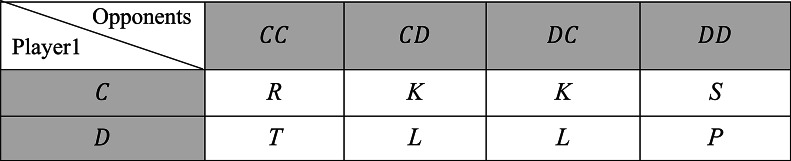
Payoff matrix in a three-player SAPD game.

$\:T$ has the highest value, whereas $\:S$ has the lowest one. Each game round displays one of the eight possible states $\:CCC,\:CCD,\:CDC,\:CDD,\:DCC,\:DCD,\:DDC$ or $\:DDD$, where the first place indicates the action taken by player 1, while the second and third places denote the options of the other two players. For instance, $\:DCC$ denotes when player 1 defects, and the other ones cooperate, then player 1 will receive $\:T$. Because of the symmetry of the game and the fact that the identities of the players are irrelevant, the outcome of player 1 who cooperates, and the other players are playing $\:CD$ or $\:DC$ is $\:K$.

According to Taha and Ghoneim^24^, we have the relations

$$\:\mathrm{T}>R>L>K>P>S\:,\:2R>T+K\:\mathrm{a}\mathrm{n}\mathrm{d}\:2K>L+S$$

Let us assume that each player considers the history of the last three successive rounds which represent one unit in alternating model. Let us consider a one unit-memory strategy used by player 1 is $\:\boldsymbol{p}=\left({p}_{\mathrm{1,2}},{p}_{\mathrm{1,1}}{,p}_{\mathrm{1,1}},{p}_{\mathrm{1,0}},{p}_{\mathrm{2,2}},{p}_{\mathrm{2,1}},{p}_{\mathrm{2,1}},{p}_{\mathrm{2,0}}\right)$, where the components of $\:\boldsymbol{p}$ represent the probabilities of cooperating after a definite outcome in the previous round. The player’s choice and the number of players who cooperate with one another in the previous round are indicated by the subscripts in each element. For instance, $\:{p}_{\mathrm{1,2}}$ indicates the probability of cooperating when the players adopt cooperation in the previous round (i.e., $\:CCC$), $\:{p}_{\mathrm{1,1}}$ is the probability of cooperating when player 1 and one other player choose cooperation in the last one (i.e., $\:CCD$ or $\:CDC$). Let us emphasize that in the stochastic strategy $\:\boldsymbol{p}$, the probabilities $\:{p}_{\mathrm{1,1}}$ and $\:{p}_{\mathrm{2,1}}$ are replicated twice. This means that player 1 will employ equal cooperation probability because the game is considered to be symmetric, even though each occurrence of $\:{p}_{\mathrm{1,1}}$ and $\:{p}_{\mathrm{2,1}}$ corresponds to a distinct history of the game.

If a strategy $\:\boldsymbol{p}$ is playing versus the strategies $\:\boldsymbol{q}=\left({q}_{\mathrm{1,2}},{q}_{\mathrm{1,1}},{q}_{\mathrm{2,2}},{q}_{\mathrm{2,1}},{q}_{\mathrm{1,1}},{q}_{\mathrm{1,0}},{q}_{\mathrm{2,1}},{q}_{\mathrm{2,0}}\right)$ for player 2 and $\:\boldsymbol{r}=\left({r}_{\mathrm{1,2}},{r}_{\mathrm{2,2}},{r}_{\mathrm{1,1}},{r}_{\mathrm{2,1}},{r}_{\mathrm{1,1}},{r}_{\mathrm{2,1}},{r}_{\mathrm{1,0}},{r}_{\mathrm{2,0}}\right)$ for player 3, then the probabilities of transition among the different phases of the game can be determined by the matrix $\:\boldsymbol{A}\left(\boldsymbol{p},\boldsymbol{q},\boldsymbol{r}\right)$:

7 $$\:\left[\begin{array}{llllllll}{p}_{\mathrm{1,2}}{q}_{\mathrm{1,2}}{r}_{\mathrm{1,2}}&\:{p}_{\mathrm{1,2}}{q}_{\mathrm{1,2}}{(1-r}_{\mathrm{1,2}})&\:{p}_{\mathrm{1,2}}(1-{q}_{\mathrm{1,2}}){r}_{\mathrm{1,1}}&\:{p}_{\mathrm{1,2}}(1-{q}_{\mathrm{1,2}})(1-{r}_{\mathrm{1,1}})&\:(1-{p}_{\mathrm{1,2}}){q}_{\mathrm{1,1}}{r}_{\mathrm{1,1}}&\:(1-{p}_{\mathrm{1,2}}){q}_{\mathrm{1,1}}(1-{r}_{\mathrm{1,1}})&\:(1-{p}_{\mathrm{1,2}})(1-{q}_{\mathrm{1,1}}){r}_{\mathrm{1,0}}&\:(1-{p}_{\mathrm{1,2}})(1-{q}_{\mathrm{1,1}})(1-{r}_{\mathrm{1,0}})\\\:{p}_{\mathrm{1,1}}{q}_{\mathrm{1,1}}{r}_{\mathrm{2,2}}&\:{p}_{\mathrm{1,1}}{q}_{\mathrm{1,1}}\left(1-{r}_{\mathrm{2,2}}\right)&\:{p}_{\mathrm{1,1}}(1-{q}_{\mathrm{1,1}}){r}_{\mathrm{2,1}}&\:{p}_{\mathrm{1,1}}(1-{q}_{\mathrm{1,1}})(1-{r}_{\mathrm{2,1}})&\:(1-{p}_{\mathrm{1,1}}){q}_{\mathrm{1,0}}{r}_{\mathrm{2,1}}&\:(1-{p}_{\mathrm{1,1}}){q}_{\mathrm{1,0}}(1-{r}_{\mathrm{2,1}})&\:(1-{p}_{\mathrm{1,1}})(1-{q}_{\mathrm{1,0}}){r}_{\mathrm{2,0}}&\:(1-{p}_{\mathrm{1,1}})(1-{q}_{\mathrm{1,0}})(1-{r}_{\mathrm{2,0}})\\\:{p}_{\mathrm{1,1}}{q}_{\mathrm{2,2}}{r}_{\mathrm{1,2}}&\:{p}_{\mathrm{1,1}}{q}_{\mathrm{2,2}}{(1-r}_{\mathrm{1,2}})&\:{p}_{\mathrm{1,1}}\left(1-{q}_{\mathrm{2,2}}\right){r}_{\mathrm{1,1}}&\:{p}_{\mathrm{1,1}}(1-{q}_{\mathrm{2,2}})(1-{r}_{\mathrm{1,1}})&\:(1-{p}_{\mathrm{1,1}}){q}_{\mathrm{2,1}}{r}_{\mathrm{1,1}}&\:(1-{p}_{\mathrm{1,1}}){q}_{\mathrm{2,1}}(1-{r}_{\mathrm{1,1}})&\:(1-{p}_{\mathrm{1,1}})(1-{q}_{\mathrm{2,1}}){r}_{\mathrm{1,0}}&\:(1-{p}_{\mathrm{1,1}})(1-{q}_{\mathrm{2,1}})(1-{r}_{\mathrm{1,0}})\\\:{p}_{\mathrm{1,0}}{q}_{\mathrm{2,1}}{r}_{\mathrm{2,2}}&\:{p}_{\mathrm{1,0}}{q}_{\mathrm{2,1}}{(1-r}_{\mathrm{2,2}})&\:{p}_{\mathrm{1,0}}(1-{q}_{\mathrm{2,1}}){r}_{\mathrm{2,1}}&\:{p}_{\mathrm{1,0}}\left(1-{q}_{\mathrm{2,1}}\right)\left(1-{r}_{\mathrm{2,1}}\right)&\:(1-{p}_{\mathrm{1,0}}){q}_{\mathrm{2,0}}{r}_{\mathrm{2,1}}&\:(1-{p}_{\mathrm{1,0}}){q}_{\mathrm{2,0}}(1-{r}_{\mathrm{2,1}})&\:(1-{p}_{\mathrm{1,0}})(1-{q}_{\mathrm{2,0}}){r}_{\mathrm{2,0}}&\:(1-{p}_{\mathrm{1,0}})(1-{q}_{\mathrm{2,0}})(1-{r}_{\mathrm{2,0}})\\\:{p}_{\mathrm{2,2}}{q}_{\mathrm{1,2}}{r}_{\mathrm{1,2}}&\:{p}_{\mathrm{2,2}}{q}_{\mathrm{1,2}}{(1-r}_{\mathrm{1,2}})&\:{p}_{\mathrm{2,2}}(1-{q}_{\mathrm{1,2}}){r}_{\mathrm{1,1}}&\:{p}_{\mathrm{2,2}}(1-{q}_{\mathrm{1,2}})(1-{r}_{\mathrm{1,1}})&\:\left(1-{p}_{\mathrm{2,2}}\right){q}_{\mathrm{1,1}}{r}_{\mathrm{1,1}}&\:(1-{p}_{\mathrm{2,2}}){q}_{\mathrm{1,1}}(1-{r}_{\mathrm{1,1}})&\:(1-{p}_{\mathrm{2,2}})(1-{q}_{\mathrm{1,1}}){r}_{\mathrm{1,0}}&\:(1-{p}_{\mathrm{2,2}})(1-{q}_{\mathrm{1,1}})(1-{r}_{\mathrm{1,0}})\\\:{p}_{\mathrm{2,1}}{q}_{\mathrm{1,1}}{r}_{\mathrm{2,2}}&\:{p}_{\mathrm{2,1}}{q}_{\mathrm{1,1}}{(1-r}_{\mathrm{2,2}})&\:{p}_{\mathrm{2,1}}(1-{q}_{\mathrm{1,1}}){r}_{\mathrm{2,1}}&\:{p}_{\mathrm{2,1}}(1-{q}_{\mathrm{1,1}})(1-{r}_{\mathrm{2,1}})&\:(1-{p}_{\mathrm{2,1}}){q}_{\mathrm{1,0}}{r}_{\mathrm{2,1}}&\:\left(1-{p}_{\mathrm{2,1}}\right){q}_{\mathrm{1,0}}\left(1-{r}_{\mathrm{2,1}}\right)&\:(1-{p}_{\mathrm{2,1}})(1-{q}_{\mathrm{1,0}}){r}_{\mathrm{2,0}}&\:(1-{p}_{\mathrm{2,1}})(1-{q}_{\mathrm{1,0}})(1-{r}_{\mathrm{2,0}})\\\:{p}_{\mathrm{2,1}}{q}_{\mathrm{2,2}}{r}_{\mathrm{1,2}}&\:{p}_{\mathrm{2,1}}{q}_{\mathrm{2,2}}(1-{r}_{\mathrm{1,2}})&\:{p}_{\mathrm{2,1}}(1-{q}_{\mathrm{2,2}}){r}_{\mathrm{1,1}}&\:{p}_{\mathrm{2,1}}(1-{q}_{\mathrm{2,2}})(1-{r}_{\mathrm{1,1}})&\:(1-{p}_{\mathrm{2,1}}){q}_{\mathrm{2,1}}{r}_{\mathrm{1,1}}&\:(1-{p}_{\mathrm{2,1}}){q}_{\mathrm{2,1}}(1-{r}_{\mathrm{1,1}})&\:\left(1-{p}_{\mathrm{2,1}}\right)\left(1-{q}_{\mathrm{2,1}}\right){r}_{\mathrm{1,0}}&\:(1-{p}_{\mathrm{2,1}})(1-{q}_{\mathrm{2,1}})(1-{r}_{\mathrm{1,0}})\\\:{p}_{\mathrm{2,0}}{q}_{\mathrm{2,1}}{r}_{\mathrm{2,2}}&\:{p}_{\mathrm{2,0}}{q}_{\mathrm{2,1}}(1-{r}_{\mathrm{2,2}})&\:{p}_{\mathrm{2,0}}(1-{q}_{\mathrm{2,1}}){r}_{\mathrm{2,1}}&\:{p}_{\mathrm{2,0}}(1-{q}_{\mathrm{2,1}})(1-{r}_{\mathrm{2,1}})&\:(1-{p}_{\mathrm{2,0}}){q}_{\mathrm{2,0}}{r}_{\mathrm{2,1}}&\:(1-{p}_{\mathrm{2,0}}){q}_{\mathrm{2,0}}(1-{r}_{\mathrm{2,1}})&\:(1-{p}_{\mathrm{2,0}})(1-{q}_{\mathrm{2,0}}){r}_{\mathrm{2,0}}&\:\left(1-{p}_{\mathrm{2,0}}\right)\left(1-{q}_{\mathrm{2,0}}\right)\left(1-{r}_{\mathrm{2,0}}\right)\end{array}\right]$$

For example, if the last round was $\:CDC,$ then the probability of transition from $\:CDC$ to $\:DCD$ is $\:(1-{p}_{\mathrm{1,1}}){q}_{\mathrm{2,1}}(1-{r}_{\mathrm{1,1}})$.

If the components of $\:\boldsymbol{p}$, $\:\boldsymbol{q}$ and $\:\boldsymbol{r}$ greater than zero, then the components of the matrix $\:A$ will be also greater than zero. Additionally, we find a unique stationary distribution $\:\boldsymbol{\pi\:}=({\pi\:}_{1},{\pi\:}_{2},{\pi\:}_{3},{\pi\:}_{4},{\pi\:}_{5},{\pi\:}_{6},{\pi\:}_{7},{\pi\:}_{8})$ where $\:{\pi\:}_{i}$ is the probability of being in state $\:i\:(i=\mathrm{1,2},\mathrm{3,4},\mathrm{5,6},\mathrm{7,8})$ when the number of rounds approaches infinity. The vector $\:\boldsymbol{\pi\:}$ is an eigenvector of the matrix $\:A$ associated with left eigenvalue 1, and the payoff of a player adopting $\:\boldsymbol{p}$ in facing with the players adopting $\:\boldsymbol{q}$ and $\:\boldsymbol{r}$ can be given by the dot multiplication $\:\boldsymbol{\pi\:}.{\boldsymbol{\varOmega\:}}_{1}$ as illustrated in Eq. (8), and $\:{\boldsymbol{\varOmega\:}}_{1}=(R,K,K,S,T,L,L,P)$ is player 1’s payoff vector.

8 $$\:{E}_{1}\left(\boldsymbol{p},\boldsymbol{q},\boldsymbol{r}\right)=R{\pi\:}_{1}+K{\pi\:}_{2}+K{\pi\:}_{3}+S{\pi\:}_{4}+T{\pi\:}_{5}+L{\pi\:}_{6}+L{\pi\:}_{7}+P{\pi\:}_{8}$$

Using the dot multiplication $\:\boldsymbol{\pi\:}.{\boldsymbol{\varOmega\:}}_{2}$ and $\:\boldsymbol{\pi\:}.{\boldsymbol{\varOmega\:}}_{3}$, the expected payoff of the other participants can also be computed, where $\:{\boldsymbol{\varOmega\:}}_{2}=(R,K,T,L,K,S,L,P)$ and $\:{\boldsymbol{\varOmega\:}}_{3}=(R,T,K,L,K,L,S,P)$ are the payoff vectors of player 2 and player 3, respectively.

## Repeated three-player SAPD game: ZD strategies

The ZD strategies provided in the preceding section will be applied to the three-player SAPD game. According to the matrix $\:A$ of the SAPD game described in Eq. (7) and Eq. (3), we have

$$\:\boldsymbol{\pi\:}.\boldsymbol{A}(\boldsymbol{p},\:\boldsymbol{q},\boldsymbol{r})\:=\boldsymbol{\pi\:}$$

9 $$\:\boldsymbol{\pi\:}\left(\boldsymbol{A}(\boldsymbol{p},\:\boldsymbol{q},\boldsymbol{r})\:-I\right)=\boldsymbol{\pi\:}{\boldsymbol{A}}^{{\prime\:}}(\boldsymbol{p},\:\boldsymbol{q},\boldsymbol{r})=0$$

where $\:{\boldsymbol{A}}^{{\prime\:}}(\boldsymbol{p},\:\boldsymbol{q},\boldsymbol{r})=\boldsymbol{A}(\boldsymbol{p},\:\boldsymbol{q},\boldsymbol{r})-I$, $\:0=(\mathrm{0,0},\mathrm{0,0},\mathrm{0,0},\mathrm{0,0})$, and $\:{\boldsymbol{A}}^{{\prime\:}}(\boldsymbol{p},\:\boldsymbol{q},\boldsymbol{r})$ is given by

$$\:\left[\begin{array}{llllllll}{p}_{\mathrm{1,2}}{q}_{\mathrm{1,2}}{r}_{\mathrm{1,2}}-1&\:{p}_{\mathrm{1,2}}{q}_{\mathrm{1,2}}{(1-r}_{\mathrm{1,2}})&\:{p}_{\mathrm{1,2}}(1-{q}_{\mathrm{1,2}}){r}_{\mathrm{1,1}}&\:{p}_{\mathrm{1,2}}(1-{q}_{\mathrm{1,2}})(1-{r}_{\mathrm{1,1}})&\:(1-{p}_{\mathrm{1,2}}){q}_{\mathrm{1,1}}{r}_{\mathrm{1,1}}&\:(1-{p}_{\mathrm{1,2}}){q}_{\mathrm{1,1}}(1-{r}_{\mathrm{1,1}})&\:(1-{p}_{\mathrm{1,2}})(1-{q}_{\mathrm{1,1}}){r}_{\mathrm{1,0}}&\:(1-{p}_{\mathrm{1,2}})(1-{q}_{\mathrm{1,1}})(1-{r}_{\mathrm{1,0}})\\\:{p}_{\mathrm{1,1}}{q}_{\mathrm{1,1}}{r}_{\mathrm{1,2}}&\:{p}_{\mathrm{1,1}}{q}_{\mathrm{1,1}}\left(1-{r}_{\mathrm{1,2}}\right)-1&\:{p}_{\mathrm{1,1}}(1-{q}_{\mathrm{1,1}}){r}_{\mathrm{1,1}}&\:{p}_{\mathrm{1,1}}(1-{q}_{\mathrm{1,1}})(1-{r}_{\mathrm{1,1}})&\:(1-{p}_{\mathrm{1,1}}){q}_{\mathrm{1,0}}{r}_{\mathrm{1,1}}&\:(1-{p}_{\mathrm{1,1}}){q}_{\mathrm{1,0}}(1-{r}_{\mathrm{1,1}})&\:(1-{p}_{\mathrm{1,1}})(1-{q}_{\mathrm{1,0}}){r}_{\mathrm{1,0}}&\:(1-{p}_{\mathrm{1,1}})(1-{q}_{\mathrm{1,0}})(1-{r}_{\mathrm{1,0}})\\\:{p}_{\mathrm{1,1}}{q}_{\mathrm{1,2}}{r}_{\mathrm{1,2}}&\:{p}_{\mathrm{1,1}}{q}_{\mathrm{1,2}}{(1-r}_{\mathrm{1,2}})&\:{p}_{\mathrm{1,1}}\left(1-{q}_{\mathrm{1,2}}\right){r}_{\mathrm{1,1}}-1&\:{p}_{\mathrm{1,1}}(1-{q}_{\mathrm{1,2}})(1-{r}_{\mathrm{1,1}})&\:(1-{p}_{\mathrm{1,1}}){q}_{\mathrm{1,1}}{r}_{\mathrm{1,1}}&\:(1-{p}_{\mathrm{1,1}}){q}_{\mathrm{1,1}}(1-{r}_{\mathrm{1,1}})&\:(1-{p}_{\mathrm{1,1}})(1-{q}_{\mathrm{1,1}}){r}_{\mathrm{1,0}}&\:(1-{p}_{\mathrm{1,1}})(1-{q}_{\mathrm{1,1}})(1-{r}_{\mathrm{1,0}})\\\:{p}_{\mathrm{1,0}}{q}_{\mathrm{1,1}}{r}_{\mathrm{1,2}}&\:{p}_{\mathrm{1,0}}{q}_{\mathrm{1,1}}{(1-r}_{\mathrm{1,2}})&\:{p}_{\mathrm{1,0}}(1-{q}_{\mathrm{1,1}}){r}_{\mathrm{1,1}}&\:{p}_{\mathrm{1,0}}\left(1-{q}_{\mathrm{1,1}}\right)\left(1-{r}_{\mathrm{1,1}}\right)-1&\:(1-{p}_{\mathrm{1,0}}){q}_{\mathrm{1,0}}{r}_{\mathrm{1,1}}&\:(1-{p}_{\mathrm{1,0}}){q}_{\mathrm{1,0}}(1-{r}_{\mathrm{1,1}})&\:(1-{p}_{\mathrm{1,0}})(1-{q}_{\mathrm{1,0}}){r}_{\mathrm{1,0}}&\:(1-{p}_{\mathrm{1,0}})(1-{q}_{\mathrm{1,0}})(1-{r}_{\mathrm{1,0}})\\\:{p}_{\mathrm{1,2}}{q}_{\mathrm{1,2}}{r}_{\mathrm{1,2}}&\:{p}_{\mathrm{1,2}}{q}_{\mathrm{1,2}}{(1-r}_{\mathrm{1,2}})&\:{p}_{\mathrm{1,2}}(1-{q}_{\mathrm{1,2}}){r}_{\mathrm{1,1}}&\:{p}_{\mathrm{1,2}}(1-{q}_{\mathrm{1,2}})(1-{r}_{\mathrm{1,1}})&\:\left(1-{p}_{\mathrm{1,2}}\right){q}_{\mathrm{1,1}}{r}_{\mathrm{1,1}}-1&\:(1-{p}_{\mathrm{1,2}}){q}_{\mathrm{1,1}}(1-{r}_{\mathrm{1,1}})&\:(1-{p}_{\mathrm{1,2}})(1-{q}_{\mathrm{1,1}}){r}_{\mathrm{1,0}}&\:(1-{p}_{\mathrm{1,2}})(1-{q}_{\mathrm{1,1}})(1-{r}_{\mathrm{1,0}})\\\:{p}_{\mathrm{1,1}}{q}_{\mathrm{1,1}}{r}_{\mathrm{1,2}}&\:{p}_{\mathrm{1,1}}{q}_{\mathrm{1,1}}{(1-r}_{\mathrm{1,2}})&\:{p}_{\mathrm{1,1}}(1-{q}_{\mathrm{1,1}}){r}_{\mathrm{1,1}}&\:{p}_{\mathrm{1,1}}(1-{q}_{\mathrm{1,1}})(1-{r}_{\mathrm{1,1}})&\:(1-{p}_{\mathrm{1,1}}){q}_{\mathrm{1,0}}{r}_{\mathrm{1,1}}&\:\left(1-{p}_{\mathrm{1,1}}\right){q}_{\mathrm{1,0}}\left(1-{r}_{\mathrm{1,1}}\right)-1&\:(1-{p}_{\mathrm{1,1}})(1-{q}_{\mathrm{1,0}}){r}_{\mathrm{1,0}}&\:(1-{p}_{\mathrm{1,1}})(1-{q}_{\mathrm{1,0}})(1-{r}_{\mathrm{1,0}})\\\:{p}_{\mathrm{1,1}}{q}_{\mathrm{1,2}}{r}_{\mathrm{1,2}}&\:{p}_{\mathrm{1,1}}{q}_{\mathrm{1,2}}(1-{r}_{\mathrm{1,2}})&\:{p}_{\mathrm{1,1}}(1-{q}_{\mathrm{1,2}}){r}_{\mathrm{1,1}}&\:{p}_{\mathrm{1,1}}(1-{q}_{\mathrm{1,2}})(1-{r}_{\mathrm{1,1}})&\:(1-{p}_{\mathrm{1,1}}){q}_{\mathrm{1,1}}{r}_{\mathrm{1,1}}&\:(1-{p}_{\mathrm{1,1}}){q}_{\mathrm{1,1}}(1-{r}_{\mathrm{1,1}})&\:\left(1-{p}_{\mathrm{1,1}}\right)\left(1-{q}_{\mathrm{1,1}}\right){r}_{\mathrm{1,0}}-1&\:(1-{p}_{\mathrm{1,1}})(1-{q}_{\mathrm{1,1}})(1-{r}_{\mathrm{1,0}})\\\:{p}_{\mathrm{1,0}}{q}_{\mathrm{1,1}}{r}_{\mathrm{1,2}}&\:{p}_{\mathrm{1,0}}{q}_{\mathrm{1,1}}(1-{r}_{\mathrm{1,2}})&\:{p}_{\mathrm{1,0}}(1-{q}_{\mathrm{1,1}}){r}_{\mathrm{1,1}}&\:{p}_{\mathrm{1,0}}(1-{q}_{\mathrm{1,1}})(1-{r}_{\mathrm{1,1}})&\:(1-{p}_{\mathrm{1,0}}){q}_{\mathrm{1,0}}{r}_{\mathrm{1,1}}&\:(1-{p}_{\mathrm{1,0}}){q}_{\mathrm{1,0}}(1-{r}_{\mathrm{1,1}})&\:(1-{p}_{\mathrm{1,0}})(1-{q}_{\mathrm{1,0}}){r}_{\mathrm{1,0}}&\:\left(1-{p}_{\mathrm{1,0}}\right)\left(1-{q}_{\mathrm{1,0}}\right)\left(1-{r}_{\mathrm{1,0}}\right)-1\end{array}\right]$$

Since we are concerned with the non-trivial solution of Eq. (10) and $\:\mathrm{det}({\boldsymbol{A}}^{\boldsymbol{{\prime\:}}})=0$, then by applying Cramer’s rule, we obtain $\:{\left({\boldsymbol{A}}^{{\prime\:}}\right)}^{-1}=\:\frac{1}{det\left({\boldsymbol{A}}^{{\prime\:}}\right)}\:Adj\left({\boldsymbol{A}}^{\boldsymbol{{\prime\:}}}\right)$, where $\:{\left({\boldsymbol{A}}^{\boldsymbol{{\prime\:}}}\right)}^{-1}$ is the invertible matrix of $\:{\boldsymbol{A}}^{{\prime\:}}$ and $\:Adj\left({\boldsymbol{A}}^{\boldsymbol{{\prime\:}}}\right)$ is the matrix of minors of $\:{\boldsymbol{A}}^{{\prime\:}}$. Applying Cramer’s rule, then.

$${\mathrm{det}}\left( {\boldsymbol{A^{\prime}}} \right)\left( {\boldsymbol{A^{\prime}}} \right)^{{ - 1}} = {\mathrm{~}}Adj\left( {\boldsymbol{A^{\prime}}} \right)$$

After simplifying, we get

10 $$\:det\left({\boldsymbol{A}}^{{\prime\:}}\right)I=Adj\left({\boldsymbol{A}}^{{\prime\:}}\right){\boldsymbol{A}}^{{\prime\:}}={0}_{8\times\:8}$$

where $\:{0}_{8\times\:8}$ is the matrix with all elements zero and $\:I$ is the identity matrix. When applying some elementary columns operations on matrix $\:{\boldsymbol{A}}^{\boldsymbol{{\prime\:}}}\left(\boldsymbol{p},\boldsymbol{q},\boldsymbol{r}\right)$ as follow (C1 + C2, C3 + C4, C5 + C6, C2 + C4, C8 + C7 and C7 + C6), then we get the following matrix

$$\:\boldsymbol{G}=\left[\begin{array}{llllllll}{p}_{\mathrm{1,2}}{q}_{\mathrm{1,2}}{r}_{\mathrm{1,2}}-1&\:{p}_{\mathrm{1,2}}{q}_{\mathrm{1,2}}-1&\:{p}_{\mathrm{1,2}}(1-{q}_{\mathrm{1,2}}){r}_{\mathrm{1,1}}&\:{p}_{\mathrm{1,2}}-1&\:(1-{p}_{\mathrm{1,2}}){q}_{\mathrm{1,1}}{r}_{\mathrm{1,1}}&\:(1-{p}_{\mathrm{1,2}})&\:(1-{p}_{\mathrm{1,2}})(1-{q}_{\mathrm{1,1}})&\:(1-{p}_{\mathrm{1,2}})(1-{q}_{\mathrm{1,1}})(1-{r}_{\mathrm{1,0}})\\\:{p}_{\mathrm{1,1}}{q}_{\mathrm{1,1}}{r}_{\mathrm{1,2}}&\:{p}_{\mathrm{1,1}}{q}_{\mathrm{1,1}}-1&\:{p}_{\mathrm{1,1}}(1-{q}_{\mathrm{1,1}}){r}_{\mathrm{1,1}}&\:{p}_{\mathrm{1,1}}-1&\:(1-{p}_{\mathrm{1,1}}){q}_{\mathrm{1,0}}{r}_{\mathrm{1,1}}&\:(1-{p}_{\mathrm{1,1}})&\:(1-{p}_{\mathrm{1,1}})(1-{q}_{\mathrm{1,0}})&\:(1-{p}_{\mathrm{1,1}})(1-{q}_{\mathrm{1,0}})(1-{r}_{\mathrm{1,0}})\\\:{p}_{\mathrm{1,1}}{q}_{\mathrm{1,2}}{r}_{\mathrm{1,2}}&\:{p}_{\mathrm{1,1}}{q}_{\mathrm{1,2}}&\:{p}_{\mathrm{1,1}}\left(1-{q}_{\mathrm{1,2}}\right){r}_{\mathrm{1,1}}-1&\:{p}_{\mathrm{1,1}}-1&\:(1-{p}_{\mathrm{1,1}}){q}_{\mathrm{1,1}}{r}_{\mathrm{1,1}}&\:(1-{p}_{\mathrm{1,1}})&\:(1-{p}_{\mathrm{1,1}})(1-{q}_{\mathrm{1,1}})&\:(1-{p}_{\mathrm{1,1}})(1-{q}_{\mathrm{1,1}})(1-{r}_{\mathrm{1,0}})\\\:{p}_{\mathrm{1,0}}{q}_{\mathrm{1,1}}{r}_{\mathrm{1,2}}&\:{p}_{\mathrm{1,0}}{q}_{\mathrm{1,1}}&\:{p}_{\mathrm{1,0}}(1-{q}_{\mathrm{1,1}}){r}_{\mathrm{1,1}}&\:{p}_{\mathrm{1,0}}-1&\:(1-{p}_{\mathrm{1,0}}){q}_{\mathrm{1,0}}{r}_{\mathrm{1,1}}&\:(1-{p}_{\mathrm{1,0}})&\:(1-{p}_{\mathrm{1,0}})(1-{q}_{\mathrm{1,0}})&\:(1-{p}_{\mathrm{1,0}})(1-{q}_{\mathrm{1,0}})(1-{r}_{\mathrm{1,0}})\\\:{p}_{\mathrm{1,2}}{q}_{\mathrm{1,2}}{r}_{\mathrm{1,2}}&\:{p}_{\mathrm{1,2}}{q}_{\mathrm{1,2}}&\:{p}_{\mathrm{1,2}}(1-{q}_{\mathrm{1,2}}){r}_{\mathrm{1,1}}&\:{p}_{\mathrm{2,2}}&\:\left(1-{p}_{\mathrm{1,2}}\right){q}_{\mathrm{1,1}}{r}_{\mathrm{1,1}}-1&\:-{p}_{\mathrm{2,2}}&\:(1-{p}_{\mathrm{1,2}})(1-{q}_{\mathrm{1,1}})&\:(1-{p}_{\mathrm{1,2}})(1-{q}_{\mathrm{1,1}})(1-{r}_{\mathrm{1,0}})\\\:{p}_{\mathrm{1,1}}{q}_{\mathrm{1,1}}{r}_{\mathrm{1,2}}&\:{p}_{\mathrm{1,1}}{q}_{\mathrm{1,1}}&\:{p}_{\mathrm{1,1}}(1-{q}_{\mathrm{1,1}}){r}_{\mathrm{1,1}}&\:{p}_{\mathrm{2,1}}&\:(1-{p}_{\mathrm{1,1}}){q}_{\mathrm{1,0}}{r}_{\mathrm{1,1}}&\:-{p}_{\mathrm{2,1}}&\:(1-{p}_{\mathrm{1,1}})(1-{q}_{\mathrm{1,0}})&\:(1-{p}_{\mathrm{1,1}})(1-{q}_{\mathrm{1,0}})(1-{r}_{\mathrm{1,0}})\\\:{p}_{\mathrm{1,1}}{q}_{\mathrm{1,2}}{r}_{\mathrm{1,2}}&\:{p}_{\mathrm{1,1}}{q}_{\mathrm{1,2}}&\:{p}_{\mathrm{1,1}}(1-{q}_{\mathrm{1,2}}){r}_{\mathrm{1,1}}&\:{p}_{\mathrm{2,1}}&\:(1-{p}_{\mathrm{1,1}}){q}_{\mathrm{1,1}}{r}_{\mathrm{1,1}}&\:-{p}_{\mathrm{2,1}}&\:\left(1-{p}_{\mathrm{1,1}}\right)\left(1-{q}_{\mathrm{1,1}}\right)-1&\:(1-{p}_{\mathrm{1,1}})(1-{q}_{\mathrm{1,1}})(1-{r}_{\mathrm{1,0}})\\\:{p}_{\mathrm{1,0}}{q}_{\mathrm{1,1}}{r}_{\mathrm{1,2}}&\:{p}_{\mathrm{1,0}}{q}_{\mathrm{1,1}}&\:{p}_{\mathrm{1,0}}(1-{q}_{\mathrm{1,1}}){r}_{\mathrm{1,1}}&\:{p}_{\mathrm{2,0}}&\:(1-{p}_{\mathrm{1,0}}){q}_{\mathrm{1,0}}{r}_{\mathrm{1,1}}&\:-{p}_{\mathrm{2,0}}&\:\left(1-{p}_{\mathrm{1,0}}\right)\left(1-{q}_{\mathrm{1,0}}\right)-1&\:\left(1-{p}_{\mathrm{1,0}}\right)\left(1-{q}_{\mathrm{1,0}}\right)\left(1-{r}_{\mathrm{1,0}}\right)-1\end{array}\right]$$

where the fourth column $\:{\stackrel{\sim}{\boldsymbol{p}}}_{1}=(-1+{p}_{\mathrm{1,2}},-1+{p}_{\mathrm{1,1}},{-1+p}_{\mathrm{1,1}},{-1+p}_{\mathrm{1,0}},{p}_{\mathrm{2,2}},{p}_{\mathrm{2,1}},{p}_{\mathrm{2,1}},{p}_{\mathrm{2,0}})$ and the sixth column $\:{\stackrel{\sim}{\boldsymbol{p}}}_{2}=(-{p}_{\mathrm{1,2}}+1,-{p}_{\mathrm{1,1}}+1,-{p}_{\mathrm{1,1}}+1,-{p}_{\mathrm{1,0}}+1,-{p}_{\mathrm{2,2}},-{p}_{\mathrm{2,1}},-{p}_{\mathrm{2,1}},-{p}_{\mathrm{2,0}})$ are controlled completely by the strategy of player 1. According to the existence of $\:{\stackrel{\sim}{\boldsymbol{p}}}_{1}$ and $\:{\stackrel{\sim}{\boldsymbol{p}}}_{2}$, player 1 has two different ways to employ ZD strategies in this game by using the fourth column or by using the sixth column of the matrix $\:\boldsymbol{G}$.

The result of these manipulations is a form for the dot multiplication of an arbitrary eight-element vector $\:\boldsymbol{f}$ with the vector $\:\boldsymbol{\pi\:}$ of the matrix $\:\boldsymbol{G}$,

11 $$\:\boldsymbol{\pi\:}\cdot\:\boldsymbol{f}\equiv\:D\left(\boldsymbol{p},\boldsymbol{q},\boldsymbol{r},\boldsymbol{f}\right)$$

As mentioned previously, the determinant $\:D\left(\boldsymbol{p},\boldsymbol{q},\boldsymbol{r},\boldsymbol{f}\right)$ in Eq. (11) has a unique property: its fourth and sixth columns are composed entirely of elements from strategy $\:\boldsymbol{p}$ only^13^. demonstrated that the expected payoff of player $\:\boldsymbol{\pi\:}.{\boldsymbol{\varOmega\:}}_{1}\:$ is equal to the determinant of a matrix formed by replacing the last column of $\:{\boldsymbol{A}}^{\boldsymbol{{\prime\:}}}$ by $\:{\boldsymbol{\varOmega\:}}_{1}$, also, we can compute it for other players 2 and 3. Due to the infinite repetition of the game, we have $\:\sum\:{\pi\:}_{i}=1$. Then $\:{E}_{1}=\boldsymbol{\pi\:}\cdot\:{\boldsymbol{\varOmega\:}}_{1}=\frac{D(\boldsymbol{p},\boldsymbol{q},\boldsymbol{r},{\boldsymbol{\varOmega\:}}_{1})}{D(\boldsymbol{p},\boldsymbol{q},\boldsymbol{r},1)}$, similarly, $\:{E}_{2}=\boldsymbol{\pi\:}\cdot\:{\boldsymbol{\varOmega\:}}_{2}=\frac{D(\boldsymbol{p},\boldsymbol{q},\boldsymbol{r},{\boldsymbol{\varOmega\:}}_{2})}{D(\boldsymbol{p},\boldsymbol{q},\boldsymbol{r},1)}$ and $\:{E}_{3}=\boldsymbol{\pi\:}\cdot\:{\boldsymbol{\varOmega\:}}_{3}=\frac{D(\boldsymbol{p},\boldsymbol{q},\boldsymbol{r},{\boldsymbol{\varOmega\:}}_{3})}{D(\boldsymbol{p},\boldsymbol{q},\boldsymbol{r},1)}$, where $\:1$ is the unit vector.

Let us consider four non-zero parameters $\:\alpha\:$, $\:\beta\:$,$\:\:\tau\:$, and $\:\gamma\:$, we investigate the relationship between the three players’ expected payoffs. Let $\:\boldsymbol{f}=\alpha\:{\boldsymbol{\varOmega\:}}_{1}+\beta\:{\boldsymbol{\varOmega\:}}_{2}+\tau\:{\boldsymbol{\varOmega\:}}_{3}+\gamma\:1$ and dividing by $\:D(\boldsymbol{p},\boldsymbol{q},\boldsymbol{r},1)$ in Eq. (11) to normalize, we get

12 $$\:\alpha\:{E}_{1}+\beta\:{E}_{2}+\tau\:{E}_{3}+\gamma\:=\frac{D(\boldsymbol{p},\boldsymbol{q},\boldsymbol{r},\alpha\:{\boldsymbol{\varOmega\:}}_{1}+\beta\:{\boldsymbol{\varOmega\:}}_{2}+\tau\:{\boldsymbol{\varOmega\:}}_{3}+\gamma\:1)}{D(\boldsymbol{p},\boldsymbol{q},\boldsymbol{r},1)}$$

According to the full control of player 1 to the fourth column of the matrix $\:\boldsymbol{G}$, he will adjust the strategy $\:\boldsymbol{p}$ to coincide with the last column of the same matrix in such a way that

13 $$\:{\stackrel{\sim}{\boldsymbol{p}}}_{1}=\left(-1+{p}_{\mathrm{1,2}},-1+{p}_{\mathrm{1,1}},-1+{p}_{\mathrm{1,1}},-1+{p}_{\mathrm{1,0}},{p}_{\mathrm{2,2}},{p}_{\mathrm{2,1}},{p}_{\mathrm{2,1}},{p}_{\mathrm{2,0}}\right)=\alpha\:{\boldsymbol{\Omega\:}}_{1}+\beta\:{\boldsymbol{\Omega\:}}_{2}+\tau\:{\boldsymbol{\Omega\:}}_{3}+\gamma\:1$$

Player 1 has the ability to adjust and fully control the strategic probabilities of the ZD strategies to ensure that the fourth column of matrix $\:\boldsymbol{G}$ coincides with the last column of that matrix. By choosing the right control parameters, player 1 can effectively limit the payoff relationships between all players and make the determinant of $\:\boldsymbol{G}$ disappear. The mathematical essence of the ZD strategies is that players unilaterally enforce a linear relationship between expected payoffs, then we get

14 $$\:\alpha\:{E}_{1}+\beta\:{E}_{2}+\tau\:{E}_{3}+\gamma\:=0$$

### Equalizer ZD strategies using the strategy $\:{\stackrel{\sim}{\boldsymbol{p}}}_{1}$

We will examine the equalizer ZD strategies when player 1 uses the fourth column, denoted by $\:{\stackrel{\sim}{\boldsymbol{p}}}_{1}$. By employing “equalizer ZD strategies” and disregarding the other players’ identities, the ZD player has the ability to enforce a fixed total expected payoff for the remaining players. By setting $\:\alpha\:=0$ and $\:\beta\:=\tau\:$ in Eq. (14), we get

15 $$\:{E}_{2}+{E}_{3}=-\frac{\gamma\:}{\beta\:}$$

By using Eq. (13) with $\:\alpha\:=0$, we get the values of $\:\gamma\:$ and $\:\beta\:$ as follows

$$\:\gamma\:=\frac{R{p}_{\mathrm{2,0}}+P(-{p}_{\mathrm{1,2}}+1)}{R-P}$$

$$\:\beta\:=\tau\:=\frac{-{p}_{\mathrm{2,0}}-\left(-{p}_{\mathrm{1,2}}+1\right)}{2(R-P)},$$

16 $$\:{E}_{2}+{E}_{3}=-\frac{\gamma\:}{\beta\:}=\frac{2R{p}_{\mathrm{2,0}}+2P(1-{p}_{\mathrm{1,2}})}{{p}_{\mathrm{2,0}}+\left(1-{p}_{\mathrm{1,2}}\right)}$$

In Eq. (16), we notice that an average of $\:2P$ and $\:2R$ with frequencies $\:(1-{p}_{\mathrm{1,2}})$ and $\:{p}_{\mathrm{2,0}}$, respectively, meaning that by employing the equalizer ZD strategy, the player may set $\:{E}_{2}+{E}_{3}$ between $\:2P$ and $\:2R$.

With the values of $\:\gamma\:$ and $\:\beta\:$, we can deduce the six components of the strategy $\:{\stackrel{\sim}{\boldsymbol{p}}}_{1}$ (note that $\:{p}_{\mathrm{1,1}}$ and $\:{p}_{\mathrm{2,1}}\:$appear twice within $\:{\stackrel{\sim}{\boldsymbol{p}}}_{1}$) from equation (13) in terms of $\:{p}_{\mathrm{2,0}}$ and $\:{p}_{\mathrm{1,2}}$, then we have

17 $$\left. {\begin{array}{*{20}{l}} {{p_{1,1}}=\frac{{\left( {{p_{2,0}}+1} \right)\left( {2R - K - T} \right)+{p_{1,2}}\left( {K+T - 2P} \right)}}{{2\left( {R - P} \right)}}} \\ {{p_{1,0}}=\frac{{\left( {R - L} \right){p_{2,0}}+\left( {L - P} \right)\left( {{p_{1,2}} - 1} \right)}}{{\left( {R - P} \right)}}+1} \\ {{p_{2,2}}=\frac{{\left( {R - K} \right){p_{2,0}}+\left( {{p_{1,2}} - 1} \right)\left( {K - P} \right)}}{{\left( {R - P} \right)}}} \\ {{p_{2,1}}=\frac{{\left( {2R - S - L} \right){p_{2,0}}+\left( {{p_{1,2}} - 1} \right)\left( {S+L - 2P} \right)}}{{2\left( {R - P} \right)}}} \end{array}} \right\}$$

Since $\:0\le\:{p}_{\mathrm{1,1}},\:{p}_{\mathrm{1,0}},\:\:{p}_{\mathrm{2,2}},\:{p}_{\mathrm{2,1}}\le\:1$, thereafter, by addressing these inequalities mathematically with Eq. (17) to find the feasible region of the equalizer ZD strategies, we obtain

18 $$\:\mathrm{m}\mathrm{a}\mathrm{x}\left\{\frac{K+T-R}{K+T-2P},\frac{L+P-2R}{L-P},\frac{2K-P-R}{K-P},\frac{2\left(S+L-R-P\right)}{S+L-2P}\right\}\le\:{p}_{\mathrm{1,2}}\le\:\mathrm{min}\left\{\frac{2P+K+T}{K+T-2P},\frac{R+K-2P}{K-P},\frac{R+S+L-3P}{S+L-2P},1\right\}\:\:\:\:\:\:\:\:\:\:\:\:\:\:\:\:\:\:\:\:\:$$

19 $$\:\mathrm{max}\left\{\frac{2\left(P-R\right)}{2R-K-T},0\right\}\le\:{p}_{\mathrm{2,0}}\le\:\mathrm{min}\left\{\frac{2P+K+T}{2R-K-T},\frac{L-P}{R-P},\frac{R+K-2P}{R-K},\frac{R+S+L-3P}{2R-S-L}\right\}\:\:\:\:\:\:\:\:\:\:\:\:\:\:\:\:\:\:\:\:$$

By selecting the values of $\:{p}_{\mathrm{1,2}}$ and $\:{p}_{\mathrm{2,0}}$, the player can compute the other components of $\:{\stackrel{\sim}{\boldsymbol{p}}}_{1}$.

Now, we will discuss the ability of player 1 to pin his expected payoff by using $\:{\stackrel{\sim}{\boldsymbol{p}}}_{1}$. In Eq. (14), we set $\:\beta\:=\tau\:=0$ and $\:\alpha\:\ne\:0$, then $\:{E}_{1}=-\frac{\gamma\:}{\alpha\:}$, and the strategy of player 1 can be determined from Eq. (13). Additionally, we computed the values of $\:\alpha\:$ and $\:\gamma\:$ to determine $\:{E}_{1}$ in relation to $\:{p}_{\mathrm{1,2}}$ and $\:{p}_{\mathrm{2,0}}$ as follows

$$\:\alpha\:=\frac{\left({p}_{\mathrm{1,2}}-1\right)-{p}_{\mathrm{2,0}}}{R-P}\:\mathrm{a}\mathrm{n}\mathrm{d}\:\gamma\:=\frac{R{p}_{\mathrm{2,0}}+P\left(-{p}_{\mathrm{1,2}}+1\right)}{R-P}$$

and

$$\:{E}_{1}=\frac{R{p}_{\mathrm{2,0}}+P\left(1-{p}_{\mathrm{1,2}}\right)}{\left(1-{p}_{\mathrm{1,2}}\right)+{p}_{\mathrm{3,0}}}$$

Using the values of $\:\alpha\:$ and $\:\gamma\:$ in Eq. (13) with $\:\beta\:=\tau\:=0$, we have

$$\:{p}_{\mathrm{1,1}}=\frac{(R-K)({p}_{\mathrm{3,0}}+1)+\left(K-P\right){p}_{\mathrm{1,2}}}{R-P}$$

$$\:{p}_{\mathrm{1,0}}=\frac{\left(R-S\right)\left({p}_{\mathrm{2,0}}+1\right)-\left(P-S\right){p}_{\mathrm{1,2}}}{R-P}$$

$$\:{p}_{\mathrm{2,2}}=\frac{\left(T-P\right)({p}_{\mathrm{1,2}}-1)+(R-T){p}_{\mathrm{2,0}}}{R-P}$$

$$\:{p}_{\mathrm{2,1}}=\frac{\left(P-L\right)(1-{p}_{\mathrm{1,2}})+(R-L){p}_{\mathrm{2,0}}}{R-P}$$

We notice that $\:0\ge\:{p}_{\mathrm{2,1}},\:{p}_{\mathrm{2,2}}$, while $\:{p}_{\mathrm{1,0}},\:{p}_{\mathrm{1,1}}\ge\:1$ and these are discarded, then the only suitable strategy is $\:\boldsymbol{p}=\left(\mathrm{1,1},\mathrm{1,1},\mathrm{0,0},\mathrm{0,0}\right)$, then player 1 is unable independently to pin his expected payoff by employing $\:{\stackrel{\sim}{\boldsymbol{p}}}_{1}$.

### Equalizer ZD strategies using the strategy $\:{\stackrel{\sim}{\boldsymbol{p}}}_{2}$

We used the previous analysis to find the equalizer strategy of player 1 when he uses the sixth column denoted by $\:{\stackrel{\sim}{\boldsymbol{p}}}_{2}$. By setting $\:\alpha\:=0$ and $\:\beta\:=\tau\:$ in Eq. (14), we get

20 $$\:{E}_{2}+{E}_{3}=-\frac{\gamma\:}{\beta\:}$$

To determine the values of $\:\gamma\:$ and $\:\beta\:$, let $\:\alpha\:=0$ in Eq. (13) after replacing $\:{\stackrel{\sim}{\boldsymbol{p}}}_{1}$ by $\:{\stackrel{\sim}{\boldsymbol{p}}}_{2}$ and solve the resulting eight equations of it, we have

$$\begin{aligned} \gamma = & \frac{{ - R{p_{2,0}} - P\left( {1 - {p_{1,2}}} \right)}}{{R - P}} \\ \beta = & \tau =\frac{{\left( {1 - {p_{1,2}}} \right)+{p_{2,0}}}}{{2\left( {R - P} \right)}} \\ \end{aligned}$$

21 $$\:{E}_{2}+{E}_{3}=-\frac{\gamma\:}{\beta\:}=\frac{2R{p}_{\mathrm{2,0}}+2P(1-{p}_{\mathrm{1,2}})}{{p}_{\mathrm{2,0}}+(1-{p}_{\mathrm{1,2}})}$$

By selecting $\:\gamma\:$ and $\:\beta\:$, we deduce the six components $\:{\stackrel{\sim}{\boldsymbol{p}}}_{2}$ from equation (13) in terms of $\:{p}_{\mathrm{2,0}}$ and $\:{p}_{\mathrm{1,2}}$, then we have

22 $$\left. {\begin{array}{*{20}{l}} {{p_{1,1}}=1 - \frac{{\left( {K+T - 2P} \right)\left( {1 - {p_{1,2}}} \right) - \left( {2R - K - T} \right){p_{2,0}}}}{{2\left( {R - P} \right)}}} \\ {{p_{1,0}}=1 - \frac{{\left( {L - P} \right)\left( {1 - {p_{1,2}}} \right)+\left( {L - R} \right){p_{2,0}}}}{{\left( {R - P} \right)}}} \\ {{p_{2,2}}=\frac{{\left( {P - K} \right)\left( {1 - {p_{1,2}}} \right)+\left( {R - K} \right){p_{2,0}}}}{{\left( {R - P} \right)}}} \\ {{p_{2,1}}=\frac{{\left( {2P - L - S} \right)\left( {1 - {p_{1,2}}} \right)+\left( {2R - S - L} \right){p_{2,0}}}}{{2\left( {R - P} \right)}}} \end{array}} \right\}$$

Since $\:0\le\:{p}_{\mathrm{1,1}},\:{p}_{\mathrm{1,0}},\:\:{p}_{\mathrm{2,2}},\:{p}_{\mathrm{2,1}}\le\:1$, then by solving the inequalities in **(22)**, we get

23 $$\:0\le\:{p}_{\mathrm{1,2}}\le\:1\:\:\:\:\:\:\:\:\:\:\:\:\:\:\:\:\:\:\:\:\:$$

24 $$\:0\le\:{p}_{\mathrm{2,0}}\le\:1\:\:\:\:\:\:\:\:\:\:\:\:\:\:\:\:\:\:\:\:$$

which means that the player can apply equalizer strategies without any restrictions on the values of $\:{p}_{\mathrm{1,2}}$ and $\:{p}_{\mathrm{2,0}}$ according to Eqs. (23) and (24) and comparing to Eqs. (18) and (19). We deduce that when selecting the value of $\:{p}_{\mathrm{1,2}}$ close to 1, then the value of $\:{p}_{\mathrm{2,0}}$ must be close to 0 and vice versa.

Now, we will discuss the ability of player 1 to pin his expected payoff by using $\:{\stackrel{\sim}{\boldsymbol{p}}}_{2}$. In Eq. (14), we set $\:\beta\:=\tau\:=0$ and $\:\alpha\:\ne\:0$, then $\:{E}_{1}=-\frac{\gamma\:}{\alpha\:}$, and the strategy of player 1 can be determined from Eq. (13) after replacing $\:{\stackrel{\sim}{\boldsymbol{p}}}_{1}$ by $\:{\stackrel{\sim}{\boldsymbol{p}}}_{2}$. Moreover, we computed the values of $\:\alpha\:$ and $\:\gamma\:$ to find $\:{E}_{1}$ in relation to $\:{p}_{\mathrm{1,2}}$ and $\:{p}_{\mathrm{2,0}}$ as follows.

$$\begin{aligned} \alpha = & \frac{{\left( {1 - {p_{1,2}}} \right)+{p_{2,0}}}}{{R - P}}{\mathrm{~and~}}\gamma =\frac{{ - R{p_{2,0}} - P\left( {1 - {p_{1,2}}} \right)}}{{R - P}} \\ {E_1}= & \frac{{R{p_{2,0}}+P\left( {1 - {p_{1,2}}} \right)}}{{\left( {1 - {p_{1,2}}} \right)+{p_{2,0}}}} \\ \end{aligned}$$

Using the values of $\:\alpha\:$ and $\:\gamma\:$ in Eq. (13) after replacing $\:{\stackrel{\sim}{\boldsymbol{p}}}_{1}$ by $\:{\stackrel{\sim}{\boldsymbol{p}}}_{2}$ with $\:\beta\:=\tau\:=0$, we get.

$${p_{1,1}}=\frac{{\left( {P - K} \right)\left( {1 - {p_{1,2}}} \right)+\left( {R - K} \right){p_{2,0}}}}{{R - P}}+1$$

$${p_{1,0}}=\frac{{\left( {P - S} \right)\left( {1 - {p_{1,2}}} \right)+\left( {R - S} \right){p_{2,0}}}}{{R - P}}+1$$

$${p_{2,2}}=\frac{{\left( {P - T} \right)\left( {1 - {p_{1,2}}} \right)+\left( {R - T} \right){p_{2,0}}}}{{R - P}}$$

$${p_{2,1}}=\frac{{\left( {P - L} \right)\left( {1 - {p_{1,2}}} \right)+\left( {R - L} \right){p_{2,0}}}}{{R - P}}$$

We notice that $\:{p}_{\mathrm{2,2}}$ is less than $\:0$, and $\:{p}_{\mathrm{1,0}}$ is greater than 1 which are discarded, and the only suitable strategy is $\:\boldsymbol{p}=\left(\mathrm{1,1},\mathrm{1,1},\mathrm{1,0},\mathrm{0,0}\right)$. We conclude player 1 is unable independently to pin his payoff by using $\:{\stackrel{\sim}{\boldsymbol{p}}}_{2}$.

### Extortionate ZD strategies using the strategy $\:{\stackrel{\sim}{\boldsymbol{p}}}_{1}$

Now, we deduce and examine the extortionate ZD strategies in the SAPD game. Press and Dyson in ^13^ investigated this type of strategy in the classical PD game, in which the ZD player receives a favorable extortion ratio of the expected payoff that surpasses $\:P$. Let us investigate this type of strategies in the SAPD game according to the Eq.

25 $$\:{\stackrel{\sim}{\boldsymbol{p}}}_{1}=\left(-1+{p}_{\mathrm{1,2}},-1+\:{p}_{\mathrm{1,1}},-1+{p}_{\mathrm{1,1}},-1+{p}_{\mathrm{1,0}},{p}_{\mathrm{2,2}},{p}_{\mathrm{2,1}},{p}_{\mathrm{2,1}},{p}_{\mathrm{2,0}}\right)=\varphi\:\left[\left({\boldsymbol{\Omega\:}}_{1}-P1\right)-\chi\:\left({\boldsymbol{\Omega\:}}_{2}+{\boldsymbol{\Omega\:}}_{3}-2P1\right)\right]$$

The parameter $\:\varphi\:>0$, and $\:\chi\:\ge\:1$ is the extortion factor. In equation (25), the player who uses these strategies tries to obtain an extortion portion over the sum of the other players’ expected payoffs. By exploring equation (25), we have

26 $$\left. {\begin{array}{*{20}{l}} {{p_{1,2}}=1 - \varphi \left( {2\chi - 1} \right)\left( {R - P} \right)} \\ {{p_{1,1}}=1 - \varphi \left[ {\chi \left( {K+T - 2P} \right) - \left( {K - P} \right)} \right]} \\ {{p_{1,0}}=1 - \varphi \left[ {\left( {P - S} \right)+2\chi \left( {L - P} \right)} \right]} \\ {{p_{2,2}}=\varphi \left[ {\left( {T - P} \right) - 2\chi \left( {K - P} \right)} \right]} \\ {{p_{2,1}}=\varphi \left[ {\left( {L - P} \right) - \chi \left( {S+L - 2P} \right)} \right]} \\ {{p_{2,0}}=0} \end{array}} \right\}$$

If the parameter $\:\varphi\:=0$, thus the single suitable strategy is $\:\boldsymbol{p}=(\mathrm{1,1},\mathrm{1,1},\mathrm{0,0},\mathrm{0,0})$, also if $\:\varphi\:<0$, the probabilities $\:{p}_{\mathrm{1,0}}$, $\:{p}_{\mathrm{1,1}}$, $\:{p}_{\mathrm{1,2}}>1$, which is discarded. From Eq. (25), we have

$$\:0<\varphi\:\le\:\frac{1}{(2\chi\:-1)(R-P)}$$

$$\:0<\varphi\:\le\:\frac{1}{\chi\:\left(T+K-2P\right)-(K-P)}$$

$$\:0<\varphi\:\le\:\frac{1}{\left(T-P\right)-2\chi\:\left(K-P\right)}$$

$$\:0<\varphi\:\le\:\frac{1}{\left(P-S\right)+2\chi\:\left(L-P\right)}$$

$$\:0<\varphi\:\le\:\frac{1}{\left(L-P\right)-\chi\:\left(S+L-2P\right)}$$

Then the parameter $\:\varphi\:$ can be specified by

27 $$0<\varphi \leqslant {\mathrm{min}}\left\{ {\frac{1}{{\left( {R - P} \right)\left( {2\chi - 1} \right)}},\frac{1}{{\chi \left( {T+K - 2P} \right) - \left( {K - P} \right)}},\frac{1}{{\left( {T - P} \right) - 2\chi \left( {K - P} \right)}},\frac{1}{{\left( {P - S} \right)+2\chi \left( {L - P} \right)}},\frac{1}{{\left( {L - P} \right) - \chi \left( {S+L - 2P} \right)}}} \right\}$$

and

28 $$\frac{1}{2}<\chi <{\mathrm{min}}\left\{ {\frac{{\left( {T - P} \right)}}{{2\left( {K - P} \right)}},\frac{{\left( {L - P} \right)}}{{\left( {L - P} \right)+\left( {S - P} \right)}}} \right\}$$

### Extortionate ZD strategies using the strategy $\:{\stackrel{\sim}{\boldsymbol{p}}}_{2}$

Similar to extortionate strategies in Sect. 6.3, we will derive the extortionate strategies for player 1 by using $\:{\stackrel{\sim}{\boldsymbol{p}}}_{2}$. We have the Eq. 

29 $$\:{\stackrel{\sim}{\boldsymbol{p}}}_{2}=\left(1-{p}_{\mathrm{1,2}},\:1-{p}_{\mathrm{1,1}},1-{p}_{\mathrm{1,1}},1-{p}_{\mathrm{1,0}},-{p}_{\mathrm{2,2}},-{p}_{\mathrm{2,1}},-{p}_{\mathrm{2,1}},-{p}_{\mathrm{2,0}}\right)=\varphi\:\left[\left({\boldsymbol{\Omega\:}}_{1}-P1\right)-\chi\:\left({\boldsymbol{\Omega\:}}_{2}+{\boldsymbol{\Omega\:}}_{3}-2P1\right)\right]$$

When exploring Eq. (29), we get

30 $$\left. {\begin{array}{*{20}{l}} {{p_{1,2}}=1 - \varphi \left( {1 - 2\chi } \right)\left( {R - P} \right)} \\ {{p_{1,1}}=1 - \varphi \left[ {\left( {K - P} \right) - \chi \left( {K+T - 2P} \right)} \right]} \\ {{p_{1,0}}=1 - \varphi \left[ {\left( {S - P} \right) - 2\chi \left( {L - P} \right)} \right]} \\ {{p_{2,2}}= - \varphi \left[ {\left( {T - P} \right) - 2\chi \left( {K - P} \right)} \right]} \\ {{p_{2,1}}= - \varphi \left[ {\left( {L - P} \right) - \chi \left( {S+L - 2P} \right)} \right]} \\ {{p_{2,0}}=0} \end{array}} \right\}$$

Let us analyze the values of the parameter $\:\varphi\:$ in Eq. (30). If $\:\varphi\:=0$, then the unique possible strategy by using the sixth column will be $\:(\mathrm{1,1},\mathrm{1,1},\mathrm{0,0},\mathrm{0,0})$. If $\:\varphi\:>0$, then $\:{p}_{\mathrm{2,2}}$ and $\:{p}_{\mathrm{2,1}}$ will be negative and this is not acceptable. If $\:\varphi\:<0$, then $\:{p}_{\mathrm{1,2}}$, $\:{p}_{\mathrm{1,1}}$ and $\:{p}_{\mathrm{1,0}}$ will be greater than 1 and this also not valid. We conclude from the previous analysis that player 1 cannot use the sixth column in the matrix $\:\boldsymbol{G}$ to extortion his opponents.

## Numerical example

Here, we will present a numerical example for 3-player SAPD game. Let us consider the values $\:T=5,\:R=4,\:L=3,\:K=2,\:P=1,$ and $\:S=0$. Then according to inequalities (18) and (19) by straight forward calculations, we have

$$\:\mathrm{m}\mathrm{a}\mathrm{x} \left\{\frac{3}{5},-2,-1,-4 \right\}\le\:{p}_{\mathrm{1,2}}\le\:\mathrm{min}\left\{\frac{9}{5},\mathrm{4,4},1\right\}\:\:\:\:\:\:\:\:\:\:\:$$

$$\:\mathrm{max}\left\{-\mathrm{6,0}\right\}\le\:{p}_{\mathrm{2,0}}\le\:\mathrm{min}\left\{9,\frac{2}{3},2,\frac{4}{5}\right\}\:\:\:\:\:\:\:\:\:\:\:$$

then we get

$$\:\frac{3}{5}\le\:{p}_{\mathrm{1,2}}\le\:1\:\&\:0\le\:{p}_{\mathrm{2,0}}\le\:\frac{2}{3}.$$

The equalizer strategy $\:\boldsymbol{p}=\left({p}_{\mathrm{1,2}},{p}_{\mathrm{1,1}}{,p}_{\mathrm{1,1}},{p}_{\mathrm{1,0}},{p}_{\mathrm{2,2}},{p}_{\mathrm{2,1}},{p}_{\mathrm{2,1}},{p}_{\mathrm{2,0}}\right)$ using the fourth column in this case will be equal to $\:\left(\frac{21}{30},\frac{25}{30},\frac{25}{30},\frac{29}{30},\frac{7}{30},\frac{11}{30},\frac{11}{30},\frac{15}{30}\right)$, according to Eq. (17) with the values $\:{p}_{\mathrm{1,2}}=0.7$ and $\:{p}_{\mathrm{2,0}}=0.5$.

Let $\:{p}_{\mathrm{1,2}}=0.9$ and $\:{p}_{\mathrm{2,0}}=0.1$according to Eqs. (23) and (24), then the equalizer strategy $\:\boldsymbol{p}=\left({p}_{\mathrm{1,2}},{p}_{\mathrm{1,1}}{,p}_{\mathrm{1,1}},{p}_{\mathrm{1,0}},{p}_{\mathrm{2,2}},{p}_{\mathrm{2,1}},{p}_{\mathrm{2,1}},{p}_{\mathrm{2,0}}\right)$ by using the sixth column in this case will be equal to $\:\left(\frac{27}{30},\frac{28}{30},\frac{28}{30},\frac{29}{30},\frac{1}{30},\frac{2}{30},\frac{2}{30},\frac{3}{30}\right)$, according to Eq. (22).

Let us determine the extortionate strategies for player 1 using the fourth column. By using Eqs. (27) and (28), we are able to determine the extortion factor $\:\chi\:$ and the positive parameter $\:\varphi\:$ by the two inequalities

$$\:\frac{1}{2}<\chi\:<\mathrm{m}\mathrm{i}\mathrm{n}\left\{\mathrm{2,2}\right\}$$

$$\:\frac{1}{2}<\chi\:<2$$

Let us assume that $\:\chi\:=1.3$ in Eq. (27), then we get

$$\:\:0<\varphi\:\le\:\mathrm{min} \left(\frac{5}{24},\frac{10}{3},\frac{5}{7},\frac{5}{31},\frac{10}{7}\right)\rightarrow\:\:0<\varphi\:\le\:\frac{5}{31}$$

Let $\:\chi\:=1.3$ and $\:\varphi\:=0.1$ in Eq. (26), we get $\:\boldsymbol{p}=\left(\mathrm{0.52,0.45,0.45,0.38,0.14,0.07,0.07,0}\right)$.

## Conclusion

The alternating PD game provides an effective framework for analyzing conflict scenarios in which players take turns making decisions, with payoffs depending on both the current player’s action and the preceding moves of others. In this study’ we examined ZD strategies in strictly alternating three-player PD game, where each player acts once per round and three consecutive rounds constitute a single memory unit. We analytically derived the feasible regions of equalizer and extortion ZD strategies for one-unit memory strategies in infinitely repeated games.

Our results show that the player who moves first can exert significant control over payoffs by employing ZD strategies. Specifically, the focal player can use the strategy $\:{\stackrel{\sim}{\boldsymbol{p}}}_{1}$ for both equalizer and extortion strategies, while $\:{\stackrel{\sim}{\boldsymbol{p}}}_{2}$ is effective only for equalizer strategies. Interestingly, these findings differ substantially from the results reported in^24^ for the two-player case. In particular, the ZD strategies associated with the sixth column of matrix $\:\boldsymbol{G}$ exhibits a distinct structure under strict alternation in the three-player framework. This difference arises because the payoff relations in the three-player game depend not only on bilateral interactions but also on the ordering of moves and the cumulative influence of the third player’s action within the cycle. Consequently, the enforcement mechanism of ZD strategies becomes more constrained but simultaneously more diverse, allowing new combinations of equalizer and extortion strategies that have no counterpart in the two-player model. These results indicate that strict alternation introduces additional coupling between player’s payoffs, revealing new equilibrium patterns and strategic dependencies that enrich the theoretical landscape of ZD control in repeated games.

For future work, we plan to generalize this framework to n-player games with n-memory units and explore the existence and behavior of ZD strategies in these more complex settings. Overall, our study provides new insights into strategic control in strictly alternating interactions and extends the analytical understanding of multi-player ZD strategies.

## Data Availability

All data generated or analyzed during this study are included in this published article.
